# Prospective Study of Blood and Tibia Lead in Women Undergoing Surgical Menopause

**DOI:** 10.1289/ehp.7005

**Published:** 2004-09-07

**Authors:** Gertrud S. Berkowitz, Mary S. Wolff, Robert H. Lapinski, Andrew C. Todd

**Affiliations:** Department of Community and Preventive Medicine, Mount Sinai School of Medicine, New York, New York, USA

**Keywords:** blood lead, bone turnover, estrogen replacement therapy, lead mobilization, tibia lead

## Abstract

Despite the dramatic decline in environmental lead exposure in the United States during the past couple of decades, concern has been expressed regarding mobilization during menopause of existing lead stored in bone. To investigate whether bone lead concentrations decrease and blood lead levels increase, we conducted a prospective study of 91 women who were scheduled to undergo a bilateral oophorectomy for a benign condition at Mount Sinai Hospital in New York City during October 1994 through April 1999. We excluded women who were younger than 30 years of age or who were postmenopausal at the time of the surgery. We observed a small but significant increase in median blood lead levels between the baseline visit and the 6-month visit (0.4 μg/dL, *p* < 0.0001), particularly for women who were not on estrogen replacement therapy (0.7 μg/dL, *p* = 0.008). No significant change was observed in blood lead values between 6 and 18 months postsurgery, nor was there evidence of significant changes in tibia lead concentrations during the follow-up period. These findings do not point to substantial mobilization of lead from cortical bone during menopause.

Although there has been a substantial decline in lead exposure in the United States during the past couple of decades (Pirkle et al. 1994), mobilization of existing lead stored in bone potentially represents an important endogenous source of exposure. Specifically, it has been hypothesized that lead may be mobilized from skeletal stores during conditions of high bone turnover, such as during menopause (Silbergeld et al. 1988). Approximately 90–95% of the total body burden of lead is retained in bone (Barry 1975; Barry and Mossman 1970), where the half-life can be several decades (Börjesson et al. 1997; Gerhardsson et al. 1993; Nilsson et al. 1991; Price et al. 1992; Rabinowitz et al. 1976). During menopause, calcium and other minerals are mobilized from bone (Pounds 1984; Bronner 1992; O’Flaherty 1992; Simons 1993). Lead is covalently bound in the mineral matrix, apparently in close chemical association with calcium and phosphate (Wittmers et al. 1988). Furthermore, lead is concentrated selectively according to the type of bone, with higher accumulations in trabecular as opposed to cortical bone (Wittmers et al. 1988; Inskip et al. 1992; Lindquist et al. 1981). It has been estimated that up to 50% of trabecular bone and 30% of cortical bone is lost during a woman’s lifetime, particularly during the early menopausal years (Lindquist et al. 1981; Heaney et al. 1978; Riggs and Melton 1986). Lead is mobilized from the bone into the blood compartment. Lead in blood can then be transferred to soft tissues, including the central nervous system, where it could affect cognitive and motor functions (Landrigan et al. 1982; Ryan et al. 1987).

Age-adjusted data from the second National Health and Nutrition Examination Survey (NHANES II, 1976–1980; Silbergeld et al. 1988), the Hispanics HANES (HHANES, 1982–1984; Symanski and Hertz-Picciotto 1995), and NHANES III (1988–1994; Nash et al. 1998) showed higher blood lead levels among postmenopausal women compared with premenopausal women. Similarly, blood lead levels were higher in postmenopausal women compared with premenopausal women in a subsample of the Nurses Health Study (Korrick et al. 2002) and two studies in Mexico City (Garrido Latorre et al. 2003; Hernandez-Avila et al. 1998). However, these studies were all based on cross-sectional data, and only two investigations (Garrido Latorre et al. 2003; Korrick et al. 2002) had any information on bone lead concentrations.

This investigation represents a longitudinal study with repeated measures of blood and tibia lead and bone mineral density (BMD) measurements among women undergoing surgically induced menopause. In addition to assessing whether there was any evidence of increased endogenous lead exposure as a result of the surgical menopause, we aimed to evaluate the effects of BMD, estrogen replacement therapy (ERT), serum ferritin, and endogenous estrogen levels on changes in blood and bone lead measurements.

## Materials and Methods

The study population was recruited from women ≥ 30 years of age who had a surgical admission or discharge diagnosis of a bilateral oophorectomy for a benign condition at Mount Sinai Hospital during October 1994 through April 1999. Excluded were women with preexisting neurologic or psychiatric diseases and any medical condition that could affect bone homeostasis. Also excluded were women who were taking corticosteroids, thyroid hormone replacement, or antiseizure medications. Women who had had no menses within the previous 6 months or fewer than nine menstrual periods within the past year were considered to be postmenopausal and were therefore not included. The final study population comprised 91 premenopausal or perimenopausal women.

The study protocol included a baseline visit before or shortly after surgery and follow-up assessments at 6 and 18 months after surgery. At the baseline visit, a structured questionnaire was administered; 25 cc blood was obtained for blood lead, serum ferritin, and hormone analysis; tibia lead concentration was determined via ^109^Cd-based K shell X-ray fluorescence (XRF) analyses; and BMD was measured by dual energy X-ray absorptiometry (DXA). The 6-month visit included all of the preceding measures except for the BMD assessment. The 18-month assessment was identical to the baseline evaluation. The research protocol was approved by the institutional review board of Mount Sinai Hospital, and written informed consent was obtained from all patients.

An attempt was made to obtain the baseline assessment of each patient before surgery. This was often not feasible because of the short lead time for the surgical admission and the fact that the decision to perform a bilateral as opposed to a unilateral oophorectomy was frequently not made until during the procedure. As a result, 58.2% had a baseline assessment before surgery, and 41.8% had the baseline assessment within 2–29 days after the procedure.

Information on covariates, such as socio-demographic characteristics; height and weight; occupational and environmental exposures; medical, gynecologic, and obstetrical history, and use of medications including ERT; physical activity, cigarette smoking, and alcohol consumption, was obtained from the questionnaire. Serum ferritin levels, which are indicative of iron stores, were determined at each visit because there is some evidence that high ferritin levels are associated with lower blood lead concentrations (Baghurst et al. 1987). Estradiol levels were assessed as an indicator of endogenous estrogen levels. Levels of follicle-stimulating hormone, which is a marker of reproductive senescence, were also assessed to verify that the patients were not postmenopausal at the time of the surgery.

Blood lead was determined using graphite furnace atomic absorption spectrophotometry with Zeeman background correction (model 4100ZL; Perkin Elmer, Norwalk, CT) using the method of Parsons (1992) at the Mount Sinai Lead Laboratory. The lead laboratory was certified by the Occupational Safety and Health Administration (OSHA) and participated in two proficiency testing programs for blood lead (Centers for Disease Control and Prevention and the Wisconsin State Laboratory of Hygiene and College of American Pathologists). OSHA certification requires that proficiency tests come within 6 μg/dL of the target value (or all-method mean) if that value is < 40 μg/dL or within 15% of the target (or all-method mean) if the value is > 40 μg/dL. During a 1-year period while these samples were being analyzed, the accuracy was within 5% or on average < 0.2 μg/dL deviation from target values for 48 proficiency test samples (analyzed in masked fashion) across a wide range of values (0–100 μg/dL). A subsample of triplet samples (baseline, 6-month, and 18-month specimens) was run on the same day in the same laboratory batch for 37 women.

The bone lead measurements were performed on the anterior, middiaphysis of the left tibia, which consists primarily of cortical bone. BMD was measured for left radius/ulna, left hip femoral neck, left hip trochanter, whole left leg (which included both the tibia and the femur), lumbar spine, and whole body. The measurements were obtained with a Hologic QDR 2000 DXA densitometer (Hologic, Bedford, MA) at the Bone Densitometry Laboratory at Mount Sinai Hospital. The scans were analyzed according to computer software protocols for each site provided by the manufacturer.

The XRF method sometimes produces negative results for low bone lead concentrations. This is because the method produces an unbiased (Todd et al. 2002) point estimate of the true concentration that oscillates, because of measurement uncertainty, around the true bone lead concentration. Other researchers (Hu et al. 1998; Kim et al. 1995) have examined the retention of the negative values in the analyses of data from epidemiologic studies and have recommended the retention of all data because alternative procedures (e.g., setting the negative values to zero or to half the value of the detection limit) introduce bias.

It is not possible to assign a specific detection limit to the XRF measurements. Each lead X ray (and the coherent scatter) peak of each *in vivo* bone lead measurement spectrum has a detection limit (defined in any one of a number of ways). There is therefore no single spectrum-based detection limit value for an individual bone lead measurement. Furthermore, there is an “instrumental detection limit,” which is usually superior to the more realistic method detection limit. In addition, there is a “system performance level” (Todd et al. 1993) and other detection limit definitions described by the International Union on Pure and Applied Chemistry (Todd et al. 2001, 2002). The XRF measurement uncertainty could be used to establish a degree of confidence in the lead concentration, but those uncertainties have been shown to underestimate the standard deviation of repeated measurements (Todd et al. 2001). Nevertheless, most of the tibia lead levels in this study could be described as at or near the method detection limit.

Because tests of normality showed that the blood lead and tibia lead values were not normally distributed (Shapiro-Wilk’s test, *p* < 0.0001 and < 0.03, respectively), medians are presented. The distributions of blood and tibia lead levels were evaluated by Wilcoxon rank sum test or, if there were more than two categories, the Kruskal-Wallis test. Covariates that were either categorical or continuous were assessed by chi-square or Student’s *t*-test, respectively. Relationships between BMD and blood or tibia lead concentrations were evaluated by Spearman’s correlation coefficient. Changes in blood and bone lead levels from baseline to 6 months, baseline to 18 months, and from 6 to 18 months were evaluated by the Wilcoxon signed rank test. Changes in blood and tibia lead levels adjusted for covariates were evaluated with multiple linear regressions.

## Results

The study population consisted of 91 premenopausal and perimenopausal women ≥ 30 years of age who were scheduled to undergo a bilateral oophorectomy for a benign condition at Mount Sinai Hospital during October 1994 through April 1999.

Among the 91 women who enrolled in the study, 71 completed the 6-month visit and 63 completed the 18-month visit. The age distribution of the 91 women was as follows: 15.4% 30–44 years of age, 53.9% 45–49 years of age, and 30.8% 50–54 years of age. With respect to race/ethnicity, 52.8% were white, 16.5% were African American, 9.9% were Hispanic, and 2.3% were Asian. The participants were generally well educated: almost 70% had received college or higher education. Regarding reproductive characteristics, 67.4% had previously been pregnant and 56.2% had previously delivered a live birth.

The proportion of women who reported ERT use was 78.9% at 6 months postsurgery and 77.8% at 18 months. The proportion of ERT users who were taking a dose of 0.625 mg was 83.9% at the 6-month follow-up and 74.5% at the 18-month assessment. Among the users, 80.8% had stayed on ERT for the period between the surgery and the 6-month visit, and 53.0% had remained on ERT during the period between surgery and the 18-month visit. Current smokers comprised 18.7% of the women, and 50.5% reported consuming one or more alcoholic drinks per week. Those who were lost to follow-up were less well educated (*p* = 0.02) and had a marginally higher body mass index (BMI; *p* = 0.06) than did those who completed the follow-up visits. Other characteristics did not differ between the two groups. Furthermore, there was no significant difference in blood lead levels at baseline for those who were lost to follow-up compared with those who remained in the study, although the former group had a somewhat higher blood lead level (3.1 μg/dL vs. 2.4 μg/dL; *p* = 0.23).

The median blood lead (2.5 μg/dL; range, 0.3–11.7 μg/dL) and tibia lead (6.0 μg/g bone mineral; range, −22.2 to 36.4 μg/g) levels were low at baseline. The median blood lead levels were not significantly different for those who had the blood drawn before (2.2 μg/dL) as opposed to after the surgery (2.6 μg/dL, *p* = 0.65). There were no significant differences in median blood lead levels or changes in the blood lead levels over time when the triplicate samples that were analyzed in the same batch were compared with the samples analyzed in separate batches. Table 1 presents the median blood lead and tibia lead levels according to selected sociodemographic and lifestyle characteristics. A significant positive association was observed between number of alcoholic drinks per week and median blood lead level. There was some suggestion that blood lead levels increased with age, decreased with increasing BMI, and were lower for women who had never smoked and those who were on ERT at 6 months, but none of these results was statistically significant. The blood lead levels for the four racial/ethnic groups were similar. No association was seen for parity (data not shown).

With respect to tibia lead, significant positive associations were observed both for current cigarette smoking and the number of alcoholic drinks per week. Tibia lead levels tended to increase with age, as expected, and tended to be lower for those on ERT at 6 months. The tibia lead levels were slightly higher for African Americans and Hispanics compared with whites or Asians.

Assessment of other potential lead-related variables such as occupations, hobbies, and residential characteristics (e.g., peeling paint) revealed no significant findings, although women who reported a hobby involving potential lead exposure, such as making jewelry or stained glass, had slightly higher blood lead levels than did those who had no such hobby (Table 2). The increased blood lead levels for those who exercised on a regular basis (> 1 hr/week) is difficult to understand, because bone turnover is generally less in women who exercise (Wolff et al. 1999). Women who had ever used herbal medicines had a borderline significant elevated blood lead level. Apart from the higher tibia lead levels among women who reported a history of hyperthyroidism, no other significant findings were observed with respect to characteristics potentially related to tibia lead levels.

As expected, significant negative declines from the baseline to the 18-month BMD assessments were seen for the lumbar spine (paired *t*-test, *p* < 0.0001), the left hip femoral bone (*p* = 0.004), and the left hip trochanter (*p* = 0.005). The decline was particularly marked for the lumbar spine for those who had not been taking ERT, but a significant decrement at this site did occur even for those who had used ERT (*p* = 0.003). There was only a slight and nonsignificant (*p* > 0.05) drop in the left whole-leg BMD between the baseline and 18-month follow-up assessment, which was limited to those who were not on ERT. However, because bone lead was measured in the left tibia, adjustment for BMD was only based on the left leg. No significant correlations (based on the Spearman correlation coefficient) were seen between left-leg BMD and blood lead values either at baseline or the 18-month follow-up or the change in blood lead levels. With respect to the correlation with tibia lead concentrations, there was a significant positive relationship between the left-leg BMD at baseline and the change in tibia lead between 0 baseline and 6 months (*r_s_* = 0.31, *p* = 0.02) but no correlation at the 18-month follow-up.

Table 3 shows the median blood and tibia lead levels at baseline and at the 6-month and 18-month follow-up visits. Two women had no blood lead determinations at baseline, and seven women did not have a tibia lead assessment at baseline. The sample sizes for the 6-month and 18-month assessments are also given in Table 3. Because not all women participated in the 6-month and 18-month follow-up assessments, changes in individual blood and tibia lead levels are of greater relevance. Table 4 summarizes the median changes over the follow-up period. It may be seen that median blood lead levels increased significantly during the first 6 months but did not change significantly between 6 and 18 months postsurgery. Although the increase during the first 6 months was greater for women who were not on ERT, both women with and without ERT experienced significant increases. Similar changes were observed for the tibia lead concentrations for all women, but none of the changes was statistically significant. The tibia lead changes according to ERT status are more difficult to interpret. There was a marginal decline in tibia lead concentrations between 6 and 18 months for women who took ERT, but not for those who did not take ERT. It should be noted that some of these results may reflect the different sample sizes.

Assessment of Spearman correlation coefficients between the change in blood lead and tibia lead showed a borderline significant result for the change in blood lead and tibia lead between baseline and 18 months (*r_s_* = −0.26, *p* = 0.05). The *r_s_* for the change in blood and tibia lead between 6 and 18 months was 0.22 (*p* = 0.10). Because the correlations went in the opposite directions, these findings are not easily interpretable.

Multiple regression analysis was used to further explore the significant increase in blood lead levels between baseline and 6 months postsurgery. Variables that were considered included blood lead at baseline, alcohol consumption, estradiol and serum ferritin levels at 6 months, tibia lead adjusted for BMD of the left leg at baseline, and change in tibia lead between baseline and 6 months adjusted for BMD. The results are summarized in Table 5. The *r* ^2^ for this model was 0.22. It may be seen that the endogenous level of estradiol at 6 months, the BMD-adjusted tibia lead level at baseline, and the change in BMD-adjusted tibia lead level between baseline and 6 months were significant predictors of the change in blood lead between baseline and 6 months. Blood lead at baseline was not significant but was included in the model because exclusion of this variable resulted in a borderline significance for estradiol (*p* = 0.06). No significant interaction was observed between ERT use and baseline tibia lead level adjusted for BMD in this model (*p* = 0.38).

## Discussion

Despite the dramatic decline in environmental lead exposure that has occurred in the United States since the 1980s, certain subgroups, such as poor innercity residents and minorities, remain more likely to have elevated levels of blood lead. Pregnant and lactating women (Gulson et al. 2003; Tellez-Rojo et al. 2002) and those undergoing menopause (Nash et al. 1998) have been identified as additional groups who may be at risk for increased blood lead levels because of potential lead mobilization during conditions of high bone turnover. To date, however, there are no published prospective studies that have assessed blood lead, bone lead, and BMD changes during these conditions. Possible increases in levels of blood lead during menopause are of concern because studies of adults have shown neuro-cognitive deficits (Muldoon et al. 1994; Payton et al. 1998) and increased blood pressure (Nash et al. 2003; Symanski and Hertz-Picciotto 1995) even at relatively low blood lead levels.

Our data suggest a slight but significant increase in blood lead between baseline and 6 months after a bilateral oophorectomy. This increase was evident both for those on ERT and those who were not on ERT, although the increase was greater for the latter group. No significant changes in blood lead levels were seen between 6 and 18 months postsurgery. With respect to tibia lead concentrations, there was some suggestion of an increase between baseline and the 6-month follow-up for those who were not on ERT therapy and a decline between 6 and 18 months for those who were on ERT therapy. Thus, these findings do not point to any substantial lead mobilization during menopause. The fact that close to 80% of the women were on ERT postsurgery may explain the findings because ERT reduces bone resorption (Prestwood et al. 2000). Alternatively, current bone lead concentrations may be sufficiently low to result in the release of only small amounts of lead into the bloodstream.

Previous studies on the effects of ERT on blood and tibia lead levels are not entirely consistent. A small cross-sectional study of blood lead concentrations among postmenopausal women either on ERT or calcium supplementation found that ERT may reduce the release of lead from bone (Webber et al. 1995). However, this was evident only for cortical (tibia) and not trabecular (calcaneus) bone, even though trabecular bone is thought to be more sensitive to estrogen declines than is cortical bone. Furthermore, ERT had no effect on blood lead concentrations in the latter study. Analysis of a subgroup from the Nurses’ Health Study (Korrick et al. 2002) found higher blood lead levels in postmenopausal women who were not taking estrogens than either premenopausal women or postmenopausal women who were using ERT. Bone lead was positively associated with blood lead only among postmenopausal women who were not using ERT, and this was true both for trabecular (patella) and cortical (tibia) bone lead. A Mexican cross-sectional osteoporosis-screening study reported that trabecular bone lead (patella) was an important predictor of blood lead in postmenopausal women both for those with a natural or surgical menopause (Garrido Latorre et al. 2003). Users of ERT also had lower blood lead levels than did nonusers in this study. In contrast, another Mexican study found significantly higher blood lead values in women with a natural compared with a surgical menopause but no difference according to ERT use (Hernandez-Avila et al. 2000). Analysis of NHANES III data for 1988–1994 showed lower blood lead levels among postmenopausal women who were current ERT users compared with past or never users (Nash et al. 1998). Two studies that also assessed BMD found no association between BMD and blood lead values (Garrido Latorre et al. 2003; Muldoon et al. 1994).

With respect to other correlates of blood lead levels, positive associations have been reported with increasing age (Hernandez-Avila et al. 2000; Korrick et al. 2002; Muldoon et al. 1994; Weyermann and Brenner 1997), cigarette smoking (Muldoon et al. 1994; Weyermann and Brenner 1997), and alcohol consumption (Korrick et al. 2002; Muldoon et al. 1994; Weyermann and Brenner 1997). Alcohol consumption was significantly associated with increased blood lead levels in our data, and nonsignificant positive trends were evident for age and cigarette smoking. Use of herbal remedies has been previously linked to lead poisoning (Centers for Disease Control and Prevention 1993; Markowitz et al. 1994). In the study by Muldoon et al. (1994) of women 65–74 years of age, moderate physical activity was related to decreased blood lead values, but more strenuous activity was associated with increased lead levels. We observed higher blood lead values among women who exercised > 1 hr/week, but our numbers were too small to detect a dose–response relationship.

Only limited data are available on characteristics influencing bone lead concentrations. In a study of tibia lead concentrations, Kosnett et al. (1994) reported positive associations with age and cigarette smoking and a negative relationship with a history of lactation. Korrick et al. (2002) found that older age and lower parity were associated with higher tibia lead but only age was related to patella lead levels. We similarly found a significant positive association between tibia lead and cigarette smoking and a positive trend with age. In addition, alcohol consumption significantly increased bone lead concentration. A history of hyperthyroidism was also a significant predictor in our study. Hyperthyroidism, which can cause bone turnover (Goldman et al. 1994), would, however, be expected to be related to higher blood but not bone lead levels.

Because there were significant declines in BMD for the lumbar spine, left hip femoral neck, and left hip trochanter (but not in the left leg or the radius/ulna), there is evidence of bone turnover in this study population. However, the possibility that a release of lead from bone with subsequent redeposition cannot be discounted because tibia lead concentrations did not change significantly over the follow-up period. Nevertheless, tibia bone lead concentrations were adjusted for left-leg BMD in the final model. Another limitation of this study is the fact that the whole-leg BMD rather than just the tibial shaft BMD was measured. Because both the tibia and the femur primarily consist of cortical bone, measurement of the whole leg should not have had any major effect on our results.

## Conclusion

We observed a small but significant increase in blood lead between the baseline assessment and the 6-month postsurgical visit, particularly for women who were not on ERT after the surgical menopause. However, no significant changes were observed for the period between 6 and 18 months, nor were there any significant changes in tibia lead concentrations postsurgery. Thus, these data do not support the hypothesis of substantial lead mobilization from cortical bone during menopause.

## Figures and Tables

**Table 1:** Baseline median blood and tibia lead levels according to sociodemographic and lifestyle characteristics among 91 women with a surgical menopause, Mount Sinai Hospital, 1994–1999.

	Blood lead			Tibia lead		
Characteristic	Median (μg/dL)	No.	*p*-Value*^a^*	Median (μg/g)	No.	*p*-Value*^a^*
Age (years)						
30–44	2.1	14		2.4	12	
45–49	2.5	47		5.7	46	
50–54	2.7	28	0.39	7.6	26	0.32
Race/ethnicity						
White	2.6	55		5.7	50	
African American	2.1	19		7.5	18	
Hispanic	2.4	12		7.2	13	
Asian	2.6	3	0.61	6.0	3	0.44
Education						
Less than high school	3.4	9		4.4	9	
High school graduate	2.5	12		8.6	12	
Some college	2.0	19		6.1	18	
College graduate	2.6	47	0.39	5.6	45	0.33
BMI (kg/m^2^)						
< 25	2.6	43		6.3	40	
25–29.9	2.2	24		6.7	24	
> 30.0	2.1	20	0.59	4.5	20	0.49
Cigarette smoking						
Never	2.2	40		4.4	39	
Ex-smoker	2.5	31		7.1	28	
Current	3.4	15	0.14	11.4	16	0.02
Alcohol consumption (drinks/week)						
0	1.9	42		3.4	42	
1–6	2.6	35		7.6	35	
> 7	3.5	9	0.001	9.5	9	0.03
Coffee consumption						
0	2.1	22		3.3	23	
1–2	2.6	40		7.5	38	
> 3	2.7	15	0.63	7.1	14	0.28
ERT at 6 months						
No	3.8*^b^*	15		10.4*^b^*	15	
Yes	3.0	56	0.15*^c^*	5.8	55	0.11*^c^*

a *p*-Value is based on the Kruskal-Wallis test, unless otherwise indicated.

b Median blood and tibia lead levels at 6 months after oophorectomy.

c *p*-Value is based on the Wilcoxon rank sum test.

**Table 2:** Baseline median blood and bone lead levels according to other potential lead-related characteristics among 91 women with a surgical menopause, Mount Sinai Hospital, 1994–1999.

	Blood lead			Bone lead		
Characteristic	Median (μg/dL)	No.	*p*-Value*^a^*	Median (μg/g)	No.	*p*-Value*^a^*
Ever had lead-related hobby						
No	2.1	40		5.2	38	
Yes	2.6	44	0.12	7.6	43	0.53
History of hyperthyroidism						
No	2.5	84		5.7	80	
Yes	3.9	3	0.32	13.1	4	0.02
Physical exercise (> 1 hr/week)						
No	2.1	28		4.7	27	
Yes	2.6	56	0.04	6.9	54	0.21
Ever used herbal medicines						
No	2.3	51		6.6	50	
Yes	2.6	31	0.05	4.8	29	0.95

a *p*-Value based on Wilcoxon rank sum test.

**Table 3:** Median blood and tibia lead levels at baseline, 6 months, and 18 months after oophorectomy by ERT status among 91 women with a surgical menopause, Mount Sinai Hospital, 1994–1999.

	Blood lead (μg/dL)		Tibia lead (μg/g)	
Follow-up period	Median (range)	No.	Median (range)	No.
All women				
Baseline	2.5 (0.3–11.7)	89	6.1 (−22.2–36.4)	84
6 months postsurgery	3.2 (0.4–12.0)	71	6.8 (−14.2–29.0)	70
18 months postsurgery	3.1 (0.5–9.1)	63	5.8 (−15.4–24.2)	62
Women on ERT				
6 months postsurgery	3.0 (0.4–12.0)	56	5.8 (−14.2–24.3)	55
18 months postsurgery	3.1 (0.5–9.1)	49	4.2 (−15.4–24.2)	46
Women not on ERT				
6 months postsurgery	3.8 (1.3–11.6)	15	10.4 (−6.9–29.0)	15
18 months postsurgery	3.2 (1.6–6.7)	14	6.9 (−4.0–19.9)	16

**Table 4:** Median changes in blood and tibia lead levels during the follow-up period by ERT status among 91 women with a surgical menopause, Mount Sinai Hospital, 1994–1999.

	Blood lead (μg/dL)			Tibia lead (μg/g)		
Follow-up period	Median change (range)	*p*-Value*^a^*	No.	Median change (range)	*p*-Value*^a^*	No.
All women						
0–6 months	0.4 (−2.0–9.6)	< 0.0001	71	1.8 (−33.3–26.3)	0.22	69
6–18 months	−0.1 (−7.6–3.9)	0.36	60	−2.2 (−18.6–22.2)	0.16	58
0–18 months	0.3 (−6.2–4.7)	0.06	63	−0.4 (–34.0–31.3)	0.86	62
Women on ERT postsurgery						
0–6 months	0.3 (−2.0–4.4)	0.003	56	0.8 (−33.3–26.3)	0.59	55
6–18 months	−0.1 (−7.6–3.9)	0.62	46	−2.8 (−18.6–22.2)	0.06	42
0–18 months	0.2 (−6.2–4.7)	0.16	49	0.1 (−34.0–31.3)	0.86	46
Women not on ERT						
0–6 months	0.7 (−0.6–9.6)	0.008	15	5.0 (−10.5–19.7)	0.08	14
6–18 months	−0.2 (−2.6–2.1)	0.31	14	2.2 (−14.0–14.9)	0.72	16
0–18 months	0.4 (−1.8–2.2)	0.21	14	−0.8 (−22.4–7.4)	0.49	16

a *p*-Value is based on the Kruskal-Wallis test.

**Table 5:** Multivariate model of blood lead changes between baseline and 6 months postsurgery among 91 women with a surgical menopause, Mount Sinai Hospital, 1994–1999.

Variable	Parameter estimate	SE	*p*-Value*^a^*	Partial *r*^2^
Intercept	1.36	0.58	0.02	—
Endogenous estradiol level at 6 months	−0.01	0.01	0.03	0.09
Blood lead at baseline	−0.17	0.12	0.16	0.04
Tibia lead at baseline adjusted for BMD*^b^*	0.07	0.03	0.008	0.13
Change in tibia lead at 0–6 months adjusted for BMD*^b^*	0.06	0.02	0.01	0.12

a Based on Student’s *t*-test.

b Tibia lead concentration multiplied by mean left-leg BMD.
